# Testing the Usability of a Software for Geospatial and Transport Modeling in Acute Stroke Service Planning

**DOI:** 10.3389/fneur.2019.00694

**Published:** 2019-06-27

**Authors:** Jessalyn K. Holodinsky, Michael J. Francis, Mayank Goyal, Michael D. Hill, Noreen Kamal

**Affiliations:** ^1^Department of Community Health Sciences, Cumming School of Medicine, University of Calgary, Calgary, AB, Canada; ^2^Hotchkiss Brain Institute, Cumming School of Medicine, University of Calgary, Calgary, AB, Canada; ^3^Department of Mechanical and Manufacturing Engineering, Schulich School of Engineering, University of Calgary, Calgary, AB, Canada; ^4^Department of Clinical Neurosciences, Cumming School of Medicine, University of Calgary, Calgary, AB, Canada; ^5^Department of Radiology, Cumming School of Medicine, University of Calgary, Calgary, AB, Canada; ^6^Calgary Stroke Program, Cumming School of Medicine, University of Calgary, Calgary, AB, Canada; ^7^Department of Medicine, Cumming School of Medicine, University of Calgary, Calgary, AB, Canada; ^8^Department of Industrial Engineering, Dalhousie University, Halifax, NS, Canada

**Keywords:** geographic visualizations, acute stroke, software, patient transport, health services research

## Abstract

**Introduction:** Geographic visualizations have been used to understand disease since the nineteenth century. We developed a software that creates simple visualizations which can be used as a decision support tool for pre-hospital acute stroke transportation planning. In this study, we test the usability of this software to improve user experience and assess the interpretability of the visualizations it produces as it relates to planning and optimizing stroke systems of care.

**Materials and Methods:** Healthcare practitioners and administrators working within the acute stroke system in Alberta, Canada were invited to participate. Participants were randomized to either the geographic visualization or 2-D temporospatial diagrams. Using a standardized script participants were asked to complete tasks and interpret the visualizations produced by the software. The computer screen and audio were recorded. Recordings were transcribed verbatim and analyzed using inductive thematic analyses. The number of errors made and time to task completion were also analyzed.

**Results:** Eighteen participants (8 physicians, 5 healthcare administrators, 3 paramedics, and 2 nurses) were enrolled. Mean age was 41.22 years (SD: 10.55) and 8 participants were female. It took users a mean of 1.59 min (SD: 0.71) to complete all 10 tasks for the geographic visualizations and a mean of 1.08 min (SD: 0.33) to complete all 15 tasks for the 2-D temporospatial diagrams. Map users made a median of 2 errors (IQR: 4), 2-D temporospatial diagram users also made a median of 2 errors (IQR: 1.5). All but one map user correctly interpreted all maps, only three of the eight 2-D temporospatial diagram correctly interpreted all diagrams. In the qualitative analysis three common themes were identified: comments on the user interface, comments on the visualization tool(s), and suggestions for improvement. Most study participants mentioned that the software would be useful in their work.

**Conclusions:** Healthcare professional from several different aspects of stroke care see geographic visualizations in transport decision making to be a useful tool. The software demonstrated high usability. However, several suggestions were made to improve user experience as well as additional features which could be developed and become the subject of future studies.

## Introduction

The use of geographic visualizations to better understand disease dates back to Dr. John Snow's seminal studies of cholera outbreak in London in the nineteenth century (1). Geographic and spatial visualizations continue to be used today, classically in infectious disease epidemiology to track disease burden in the population, assess patterns of disease transmission, and predict or manage disease outbreaks (2–8). However, geographic visualizations can now be found in cancer epidemiology (9), the planning of emergency room and neonatal intensive care unit locations (10, 11), as well as analyzing equity in access to healthcare services (12–14).

The consideration of geography is especially timely and important in stroke care right now. The complex pre-hospital decision making required for optimal transportation of acute ischemic stroke patients with suspected large vessel occlusion (LVO) lends itself well to geographic visualizations and analyses. For patients with acute ischemic stroke due to suspected LVO endovascular therapy (EVT) was recently proven both more effective than and synergistic with alteplase (15–19). However, EVT is typically only available at large urban hospitals (herein referred to as EVT centers) while alteplase is more widely available at EVT centers and at smaller community hospitals (herein referred to as thrombolysis centers). There are two transport options available to these patients: (1) direct transport to an EVT center for alteplase and/or EVT, which may entail bypassing closer thrombolysis centers (mothership transport); or (2) transport to a thrombolysis center for alteplase first and then transfer to an EVT center for EVT (drip-and-ship transport). Given the geographic disparity in treatment availability and the highly time-sensitive nature of both treatments the optimal transport method is often unknown.

**Figure 1:**
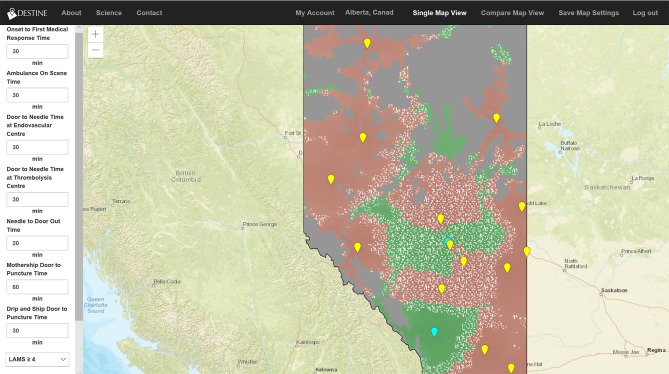
Generated map from the DESTINE software (v0.01) showing Alberta, which was used in the usability tests. The user data entry panel is shown on the left-hand side. The top bar contains clickable items allowing the user to select the region of interest as well as convert from single map view to the compare map view. The resultant map visualization is displayed in the middle of the screen. The maps display endovascular therapy centers as blue icons and thrombolysis centers as yellow icons. The maps are color coded such that areas in red indicate that drip-and-ship predicts best patient outcomes, areas in green indicate mothership predicts best outcomes, and areas overlaid with white stippling indicate the difference in probability of good outcome between the two options is minimal (<0.01).

**Figure 2:**
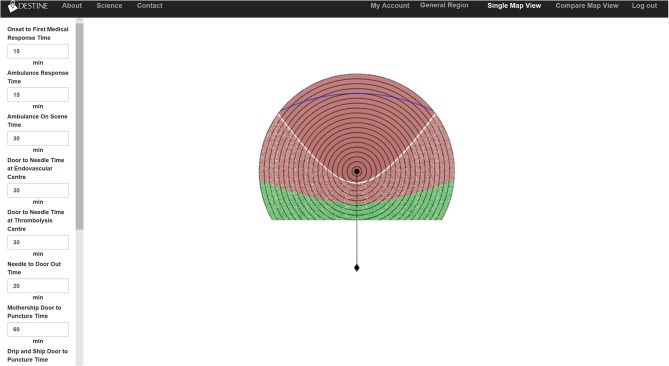
Two-dimensional temporospatial diagrams from the DESTINE software (v0.01), which was used in the usability tests. The user data entry panel is shown on the left-hand side. The top bar contains clickable items allowing the user to select the region of interest as well as convert from single view to the compare view. The resultant temporospatial visualization is displayed in the middle of the screen. In the visualizations the thrombolysis center is represented by a black dot and the EVT center is represented by a black diamond below it. The travel time between the two centers is represented by the vertical line connecting them. The thrombolysis center is surrounded by concentric circles representing 5-min increments of travel time. The concentric circles only extend to the half-way point between the two centers as below this point the EVT center is the closest hospital to the patient and decision making would not be needed. The diagram is color coded such that areas in red indicate that drip-and-ship predicts best patient outcomes, areas in green indicate mothership predicts best outcomes and areas overlaid with white stippling indicate the difference in probability of good outcome between the two options is minimal (<0.01).

Previously, a conditional probability model which determines the best transport option (drip-and-ship or mothership) based on predicted patient outcomes has been developed (20). Due to the complexity of the model a simple geographic visualization was needed to clearly display its results. A cloud-based software application [DEcision Support Tool IN Endovascular therapy (DESTINE)] was developed to display the models results visually (21). The software is customizable to a given region's unique circumstances (location of treatment centers, treatment efficiency, and field population). The software can generate maps of specific geographies (see example in Figure 1) or generalized figures (2-dimensional temporospatial diagrams) for a non-specific regions (see example in Figure 2) which depict the best transport decision for patients.

As the use of geography in clinical decision making grows there is a need to develop easy to use software programs to convey this information both quickly and simply to decision makers. It is important that in parallel with the development of decision support tools their usability be tested. The purpose of performing usability studies is to ensure that products are easy to learn, effective to use, and users have a positive experience with the product (22). Usability study can involve several different methodologies. Here we employ feature inspection to assess usability which is the assessment of sequences to accomplish a particular task. In performing feature inspection one checks for long or unnatural sequences or steps which would require extensive experience with the product to perform (23). In this study we use feature inspection to assess the speed and ease of software use and identify any areas for improvement. As the 2-D temporospatial diagrams are a novel visualization technique and the use of maps visualizations as a decision support tool for stroke transportation is also new we also aimed to assess the interpretability of these visualizations in planning and optimizing stroke systems of care.

## Materials and Methods

### Participants

Healthcare practitioners and administrators working within the acute stroke system in Alberta, Canada were invited to participate. Users from all aspects of acute stroke care spanning emergency medical services, to in-patient physicians to stroke system planners were invited to ensure a variety of perspectives were obtained.

### Usability Testing

Participants were randomized to either the geographic visualization of the province of Alberta, Canada or the 2-D temporospatial diagram. The participants were advised only that the usability of the software was being assessed, not their individual abilities. During the usability test, participants were provided with access to the software and given specific task to perform to generate visualizations. An example of a task would be: “For the map on the right, change the Door-to-Needle time at the thrombolysis center to 70 min.” The participants were then asked to interpret these visualizations (example: “In the town of Stettler, which is located just East of Red Deer, which transport option predicts the best outcome for the average patient in this scenario?”) and finally were asked several open-ended questions to garner their qualitative feedback on their user experience (example: “What additional features, if any, would you like to see?”). During the study, both the computer screen and room audio were recorded. A script was used by the researcher to ensure that each participant received the same instructions (see Supplemental File 1 for full script with all tasks and interpretation questions).

### Data Analysis

Each video and audio recording was analyzed and time to task completion and number of errors made during task completion were recorded. An error during task completion was defined as any event that the user clicked on an incorrect part of the software that would not result in the successful completion of the task. Quantitative data were tabulated and summarized using means and standard deviations (SD) or medians and interquartile ranges (IQR). Relationships between number of errors made and task length was examined using Pearson's correlation coefficient. All quantitative analyses were performed using Stata version 15.1 (Stata Corp. College Station, TX). Participant's answers to the questions regarding visualization interpretation and software user experience were transcribed verbatim by the researchers. Transcripts were read and analyzed using inductive thematic analyses. Common items were identified and organized into themes and subthemes. This process was iterated until no further themes/subthemes were identified and all comments were sorted. Qualitative data analyses were performed using Nvivo 12 (QSR International Ltd. Melbourne, AUS).

### Ethics

This study was reviewed and approved by the Conjoint Health Research Ethics Board at the University of Calgary.

## Results

### Participant Characteristics

The study enrolled 18 participants of whom 8 were physicians (including neurologists, fellows, and emergency room physicians), 5 were healthcare administrators (ranging from EMS administration to local hospital administration to provincial health network administration), 3 were paramedics, and 2 were nurses. The mean age of participants was 41.2 years (standard deviation 10.5 years) and 8 of participants were female. Participants self-reported a median computer proficiency score of 7 (IQR 1.75) on a 10-point scale with higher scores indicating better proficiency. Ten participants were randomized to evaluation of geographic visualizations of the province of Alberta, Canada; however, recording data from one of the participants was corrupted, so only data from nine participants were analyzed. Eight participants were randomized to evaluation of the 2-dimensional temporospatial diagrams. A breakdown of participant characteristics by visualization randomized to is shown in Table 1.

**Table 1:** Participant characteristics.

**Characteristic**	Randomized to Alberta map visualization (*N* = 10)^*^	**Randomized to 2-D temporospatial diagram (*N* = 8)**
Mean age (SD)	41.10 (12.11)	41.38 (9.04)
Percent female (*N*)	30% (3)	62.5% (5)
Profession		
Physician (%)	40% (4)	50% (4)
Paramedic (%)	10% (1)	25% (2)
Health administrator (%)	40% (4)	12.5% (1)
Nurse (%)	10% (1)	12.5% (1)
Median self-reported computer proficiency (IQR)	7 (1.75)	7.5 (1.75)

* *Includes demographic data from corrupted video recording*.

### Quantitative Task Performance for Geographic Visualization

Participants randomized to the geographic map visualization completed a total of 90 tasks. It took users a mean of 1.59 min (SD 0.71 min) to complete all 10 tasks. The maximum time it took a single user to complete all 10 tasks was 3.06 min. The mean time that it took users to complete each task was 9.51 s (SD: 8.05 s). The maximum time it took a single user to complete a single task was 112 s. The task that took the longest amount of time was selecting a LVO screening tool (mean: 28.33 s; SD: 33.47 s) followed by adjusting the transparency setting on the map (mean: 18.89 s; SD: 9.48 s).

Overall participants made very few errors during task completion (median: 2; IQR: 4); no participants completed all 10 tasks error free. Four of the tasks were completed error free by all participants. For the other six tasks >50% of participants completed each task error free and those who did make errors made a median of 1–2 errors per task. The maximum number of errors made by a single participant on a single task was 4 and the maximum number of errors made by a single participant across all tasks was 8. The two tasks which had the most errors made were selecting a screening tool (median: 2; IQR: 0.5; among participants who made errors) and adjusting the maps transparency (median: 2; IQR: 0.75; among participants who made errors). There was a positive correlation between mean number of errors made per task and mean length of time it took to complete the task (*r* = 0.8373, *p* = 0.0025).

### Quantitative Task Performance for 2-Dimensional Temporospatial Diagrams

The users randomized to the 2-dimensional temporospatial diagrams completed 119 of 120 tasks in total; in one instance a software error occurred and the visualization was not generated, thus the following task could not be completed. It took users an average of 1.08 min (SD 0.33 min) to complete 15 tasks. The maximum time it took a single user to complete all 15 tasks was 1.77 min. The mean time that it took users to complete each task was 5.87 s (SD: 3.38 s). The maximum time it took a single user to complete a single task was 30 s. The task that took the longest amount of time was selecting a LVO screening tool (mean: 12.75 s; SD: 5.47 s).

Overall participants made very few errors during task completion (median: 2; IQR: 1.5); one participants completed all 15 tasks error free. Ten of the tasks were completed error free by all participants. For the other five tasks >50% of participants completed each task error free and those who did make errors made a median of 1–2.5 errors per task depending on the task. The maximum number of errors made by a single participant on a single task was 3 and the maximum number of errors made by a single participant across all tasks was 4. The task which had the most errors made was selecting the generate button (median: 2.5; IQR: 0.5; among participants who made errors). Mean time to task completion was positively correlated with mean number of errors made (*r* = 0.6796, *p* = 0.0053).

### Interpretation of Map Visualizations

Users evaluating the map visualizations were asked three questions which involved interpreting the generated maps. Eight of the nine participants correctly answered all three questions which required interpretation of the visualization. One participant was only able to correctly answer one of the three interpretation questions.

### Interpretation of 2-Dimensional Temporospatial Diagrams

Users evaluating the 2-dimensional temporospatial diagrams were asked four questions which involved interpreting the generated diagram. Only three of the eight participants correctly answered all four questions which required interpretation of the diagram. Three participants were able to answer three of the four questions correctly, and two participants were able to answer two of the four questions correctly. The most incorrect answers were given on the first question asked (4/8 participants answering incorrectly), as the questions advanced less errors were made and all participants correctly answered the fourth interpretation question.

### Qualitative Analysis of User Experience

Using inductive thematic analysis three common themes (with 11 subthemes) were identified: comments on the user interface, comments on the visualization tool(s), and suggestions for improvement (Table 2). The number of times each subtheme was mentioned and the number of participants who mentioned each subtheme is shown in Table 2. All but three subthemes appeared in both the geographic map and 2-dimensional temporospatial diagram users.

**Table 2:** Common themes from inductive thematic analysis.

**Common themes with subthemes**	**Geographic map visualizations (*****N*** **=** **9)**		**2-dimensional temporospatial diagrams (*****N*** **=** **8)**	
	**Number of times theme mentioned**	**Number of users who mentioned theme**	**Number of times theme mentioned**	**Number of users who mentioned theme**
**1. Comments on user interface**				
1.1. Complexity of data entry				
1.1.1 Positive	2	2	2	2
1.1.2 Negative	3	2	7	4
1.2. Ease of use	5	4	9	4
1.3. Liked compare view feature	0	0	5	5
**2. Comments on visualization tool(s)**				
2.1 Map opacity negatively effecting readability	9	6	N/A	N/A
2.2. Visualization simplicity				
2.2.1. Simple	1	1	3	2
2.2.2. Too complex	3	1	5	3
2.3 Mentioned visualization being informative	13	6	2	2
2.4 Would use this tool in their work	8	8	7	6
**3. Suggestions for Improvement**				
3.1 Auto population of data or having real time data updates	11	7	12	4
3.2 Addition of helicopter or fixed wing air ambulance	6	4	2	1
3.3 Suggested making map area searchable	9	4	N/A	N/A
3.4 Improve map loading speed	15	6	1	1

Among comments on the software's user interface, the complexity of data entry was mentioned most frequently (five times by four geographic map users and nine times by five 2-dimensional temporospatial diagram users) (data entry panel is shown in Figure 1). This complexity was mentioned in positive and negative comments, sometimes by the same user. The majority of the comments were negative referencing both the length of time data entry would take “*I just wonder if there's a better way if you have to fill out the whole form. It's a bit of tough read and try and do quickly,”* that “*some of the fields are very similar and that was confusing,”* and “*the user may not know all these things.”* However, other participants mentioned that having a large number of variables to enter or adjust was a positive aspect. Approximately half of the participants in each group commented that the software was easy to use and five of the eight 2-dimensional temporospatial diagram users commented that they liked being able to compare the visualizations side by side. None of the map users made this comment although this feature was also available to them.

**Figure 3:**
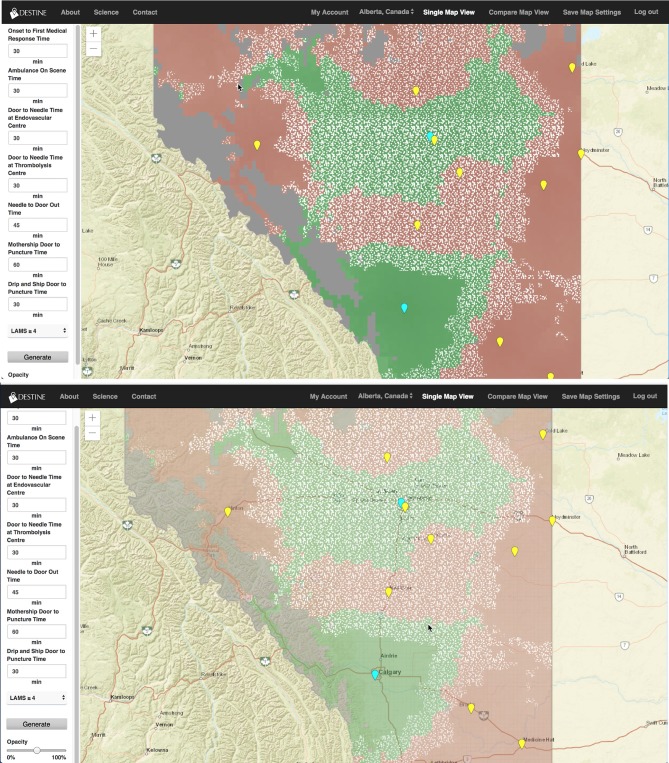
Generated map from the DESTINE software (v0.01) showing Alberta, which was used in the usability tests. The top panel shows the map generated with 100% color coding opacity and the bottom panel shows the map generated with 50% color coding opacity so that the underlying major road structures and city names can be seen. The slider bar which changes the color coding opacity is shown in the bottom left corner of the bottom panel.

Several comments were made on the visualization tools themselves. Six of the nine map users mentioned that the opaque color coding hindered their ability to find exact locations on the map and would prefer either a semi-transparent color coding system to allow geographic features to be seen underneath or for road infrastructure and city/town names to be on the top layer of the map. Figure 3 displays a side by side comparison of the same map with color coding set to 100% opacity (top panel) and 50% opacity (bottom panel).

“*The primary thing I would like to see if I was using the software is that the opacity is at 50% always or if you had another layer where the roads cities and towns were in front always.”*

There was disagreement among the users if the visualizations were simple and clear or if they were too complex (Table 2). Six of nine map users and two of eight 2-dimensional temporospatial diagram users mentioned the visualizations being informative and eight of nine map users and six of eight 2-dimensional temporospatial diagram users mentioned they or their colleagues would use this tool in their work. As illustrated in the two quotes below the use of this tool was viewed differently by those in different aspects of acute stroke care.

“*I think there is an application for EMS [emergency medical services] for sure. Part of our destination protocols is where I see this working. It's significantly better than what we use now for sure. We are making a best-case judgement on where to take people but there is a lot of factors that affect the treatment people get especially in a rural centre but if you had a way to know the numbers … this could really lead you to the right place for sure.”—Paramedic*

“*…with this condition [stroke] it's very specific and it think this would help with infrastructure planning and planning in general. The potential to use tools like this for other conditions as well would be fascinating. Absolutely, I would use it. It takes a lot of complex information and it helps us visualize some of the terms for future planning…To have something like this …you can weigh your options, current performance, improved performance, and then even with improved performance, is there still a gap or a need in the area for more infrastructure?”—Health Care Administrator*

The final theme that emerged was suggestions for improvement of the software and/or visualizations. The most common suggestion made was to auto populate certain data fields and/or have data updating in real time (mentioned a total of 23 times by 11 users). “*Having that ability for real time pre-populated info—as you know time is everything for this [stroke].”* Many users also mentioned improvements to map loading speeds would be beneficial. A common suggested addition to the visualization was the option to view helicopter or fixed wing air ambulance as a transport option for the patient (mentioned eight times by five participants). Lastly, four participants mentioned that making the map area searchable would improve the user experience.

## Discussion

This study displayed the usability of a novel visualization software for acute stroke pre-hospital transportation decision making. Both the geographic visualization and the 2-D temporospatial diagrams were relatively easy to use, as all participants were able to complete all tasks with minimal errors in a relatively short amount of time, with no prior training. There was significant variability in time to complete the study tasks among the participants (range: 53 to 184 s in the geographic visualization group and 42–106 s in the 2-D temporospatial diagram group). Time to complete tasks was significantly correlated with errors made in both study groups and as such we expect if users either received training on software operation or were allowed more time to self-learn the software the number of errors and therefore time to complete tasks would decrease. However, the group of participants had high self-rated computer proficiency which could influence these results; it is unknown if the software ease of use would be the same for users with lower computer proficiency.

In terms of interpretability the map visualizations may be more easily interpretable than the 2-D temporospatial diagrams (89 vs. 37.5% of participants correctly answering all interpretation questions). However, the intent of this study was not to compare the two visualization methods so further research would be needed to evaluate this. It was found that during the study as the questions advanced participants were able to make correct interpretations more often, thus with training and increased software familiarity we expect the interpretability of the visualizations to increase.

The majority of study participants felt that the software would be helpful for their work. As mentioned specifically by some participants in health care administration and paramedic roles this software could be used to develop acute stroke transport protocols through a more evidence-based tool that models this complex problem into a simple visualization. This is consistent with a case study that was previously performed with healthcare administrators using similar geographic visualizations using this model (but not using the software itself) (21). In the future additional studies could be performed on the impact of integration of a software such as this into decision making processes.

This study has allowed us to understand features which could be added to or changed in the software to improve the user experience. Based on feedback from the participants, two of these features are the addition of a search feature to search specific map locations as well as a more user-friendly way to change the transparency of the color visualization so that the map below it is more visible. Other items identified by the participants including the addition of air transport options and data pre-population will be the subject of future research.

There are some limitations with this study. Primarily, most 2-D temporospatial diagram participants when given free-time to use the software, found the mapping feature of the software on their own and explored it, as such some of their comments may be influenced by their use of the map view. Conversely, the opposite scenario never happened, no map participants explored the 2D temporospatial diagrams on their own. The study was performed on a relatively small number of individuals. Repeating the study in a larger sample may reduce variability in some of the quantitative measures, however in the qualitative analyses idea saturation was very quickly reached so it is unlikely that more participants would significantly impact these results. Additionally, the study could be repeated with participants in a different geographic setting to further validate the results. Finally, these results show the first use of the DESTINE software by a variety of healthcare professionals. It is anticipated that there will be a learning effect as users spend more time with the software. A follow-up study with long-time users of DESTINE would reveal additional information about the software's usability and additional features required.

Based on the results of this study healthcare professional from several different aspects of stroke care see geographic visualizations in stroke transport decision making to be a useful tool. The proposed usages varied based on healthcare professional type. The software demonstrated high usability as all participants were able to complete all tasks in a reasonable amount of time. However, several suggestions were made to improve user experience as well as additional features which could be developed and become the subject of future studies.

## Data Availability

The datasets generated for this study are available on request to the corresponding author.

## Ethics Statement

This study reviewed and approved by the Conjoint Health Research Ethics Board at the University of Calgary with written informed consent from all subjects. All subjects gave written informed consent in accordance with the Declaration of Helsinki.

## Disclosure

JH and NK hold the copyright for the DESTINE software. Information on software use and licensing can be obtained on www.destinehealth.com.

## Author Contributions

JH and NK conceived and designed the study, performed and interpreted analyses, and wrote the manuscript. MF conducted the usability sessions and assisted in data analysis. MG and MH edited the manuscript for important intellectual and clinical content.

### Conflict of Interest Statement

JH and NK report equity ownership in DESTINE Health Inc. The remaining authors declare that the research was conducted in the absence of any commercial or financial relationships that could be construed as a potential conflict of interest.
