# Supplementary material for: Testing the Usability of a Software for Geospatial and Transport Modeling in Acute Stroke Service Planning
Source: Front Neurol. 2019 Jun 27;10:694. doi: 10.3389/fneur.2019.00694 (PMC6610475; doi:10.3389/fneur.2019.00694)
Supplement: Supplementary file 1 [file Data_Sheet_1.pdf]

Supplementary Online Content

Testing the Usability of a Software for Geospatial and Transport Modelling in Acute Stroke Service Planning

Jessalyn K. Holodinsky, MSc Michael J. Francis, BSc (In Progress), Mayank Goyal, MD, Michael D. Hill, MD MSc, and Noreen Kamal, PhD

Supplemental File 1. DESTINE Usability Study Script with Tasks and Questions

DESTINE Usability Study Script with Tasks and Questions

1. Geographic visualization script

Introduction Script:
"Welcome to the DESTINE usability study. Your participation is greatly appreciated. The DESTINE software produces maps which show whether the drip-and-ship or mothership transport method predicts the best outcome for stroke patients suspected to have a large vessel occlusion given various factors including the location of the patient and treatment times at nearby hospitals. The overall purpose of the software is to assist in the design and to optimize acute stroke systems of care. When a map is produced, areas in red indicate drip-and-ship is best and areas in green indicate mothership is best. The stippled areas show when either transport option yields similar a probability of good outcome (+ or – 1% of each other)."

"The first part of this study is to see how a user completes specific tasks. We will provide you with specific tasks and you will need to complete them. The screen will be recorded and we will be recording audio as well. We will use the recordings to determine how long it took to complete a task and the number of errors that were made. This is not a test of your abilities it is only a test of the software's design and features. Do you feel comfortable to continue?"

"The computer in front of you has been logged into the DESTINE website using a test account, you are now on the home page of the website"

"Task: Select Map"

"Task: Select the Alberta Region."

"Task: Select the field screening tool of LAMS>= 4"

"Task: Change the Needle-to-Door-Out time to 45 minutes."

"Task 5: Generate the map."

"Task: Click on any region with a red or green colour to see what the probability of good outcome is."

"Task: Reduce the transparency of the colour to see the map below."

"Task: Zoom in on the map to view the city of Edmonton"

"Task: Zoom out on the map to view the entire province of Alberta"

"Question: In the town of Stettler, which is located just East of Red Deer, which transport option predicts the best outcome for the average patient in this scenario?"

"Task: Now you want to compare this map to a different scenario. Select compare map."

“Task: For the map on the right, change the Door-to-Needle time at the thrombolysis centre to 70 minutes.”

“Task: Leave all parameters as is and generate the map on the right.”

“Question: What key difference do you observe between the map on the left and the map on the right? Does Stettler still show the same transport option? What else do you observe?”

“Please take some time now to use the software as you want. We feel 15 minutes is enough time, but let us know if you require more time. Once you have had enough time to fully familiarize yourself with the software, we have some final question for you.”

“Question: What are your overall impressions of the software?”

“Question: What additional features, if any, would you like to see?”

“Question: What did you like about the software?”

“Question: What did you dislike about the software?”

“Question: If you had easy access to this tool would you see it as being useful in your work? If so how?”

“Question: Do you have any additional thoughts that you would like to share?”

2. 2-D TEMPORAL SPATIAL VISUALIZATION

Introduction Script:
"Welcome to the DESTINE usability study. Your participation is greatly appreciated. The DESTINE software produces visualizations which show whether the drip-and-ship or mothership transport method predicts the best outcome for stroke patients suspected to have a large vessel occlusion given various factors including the location of the patient and treatment times at nearby hospitals. The overall purpose of the software is to assist in the design and to optimize acute stroke systems of care. When a visualization is produced, areas in red indicate drip-and-ship is best and areas in green indicate mothership is best. The stippled areas show when either transport option yields similar a probability of good outcome (+ or – 1% of each other)."

"The first part of this study is to see how a user completes specific tasks. We will provide you with specific tasks and you will need to complete them. The screen will be recorded and we will be recording audio as well. We will use the recordings to determine how long it took to complete a task and the number of errors that were made. This is not a test of your abilities it is only a test of the software's design and features. Do you feel comfortable to continue?"

"The computer in front of you has been logged into the DESTINE website using a test account, you are now on the home page of the website"

"Task: Select Map"

"Task: Select the General Region."

"Task: Select the field screening tool of LAMS>= 4"

"Task: Change the Needle-to-Door-Out time to 45 minutes."

"Task: Generate the visualization."

"Each concentric circle represents a time of 5 minutes. The thrombolysis centre is the circle and the EVT centre is the diamond"

"Question: How many minutes below the thrombolysis centre do you have to be for Mothership to be the best transport option?

"Question: How many minutes below the thrombolysis centre do you have to be for either Drip and Ship or Mothership to yield the same probability of good outcome?"

"Task: Generate a graphic that would be similar to [HOSPITAL NAME] as the thrombolysis centre and [HOSPITAL NAME] to be the EVT centre. Here are the variables:

- Time between thrombolysis and EVT centre is 150 minutes
- Time from onset to first medical contact is 30 minutes

- On-Scene time is 20 minutes
- DTN at thrombolysis scentre is 30 minutes
- Needle to door out time is 30 minutes
- DTN at EVT centre is 35 minutes
- Door-to-groin puncture time is 60 minutes

"Question: At which time point does mothership become better than drip and ship?"

"Task: Now you want to compare this visualization to a different scenario. Select compare."

"Task: For the map on the right, change the Door-to-Needle time at the thrombolysis centre to 70 minutes."

"Task: Leave all parameters as is and generate the map on the right."

"Question: What key difference do you observe between the map on the left and the map on the right?

"Please take some time now to use the software as you want. We feel 15 minutes is enough time, but let us know if you require more time. Once you have had enough time to fully familiarize yourself with the software, we have some final question for you."

"Question: What are your overall impressions of the software?"

"Question: What additional features, if any, would you like to see?"

"Question: What did you like about the software?"

"Question: What did you dislike about the software?"

"Question: If you had easy access to this tool would you see it as being useful in your work? If so how?"

"Question: Do you have any additional thoughts that you would like to share?"
